# Intrapartum antibiotics for GBS prophylaxis alter colonization patterns in the early infant gut microbiome of low risk infants

**DOI:** 10.1038/s41598-017-16606-9

**Published:** 2017-11-28

**Authors:** Jennifer C. Stearns, Julia Simioni, Elizabeth Gunn, Helen McDonald, Alison C. Holloway, Lehana Thabane, Andrea Mousseau, Jonathan D. Schertzer, Elyanne M. Ratcliffe, Laura Rossi, Michael G. Surette, Katherine M. Morrison, Eileen K. Hutton

**Affiliations:** 1 Department of Medicine, McMaster University, Hamilton, Canada; 2 Farncombe Family Digestive Health Research Institute, McMaster University, Hamilton, Canada; 3 Midwifery Education Program, McMaster University, Hamilton, Canada; 4 Department of Pediatrics, McMaster University, Hamilton, Canada; 5 Department of Obstetrics & Gynecology, McMaster University, Hamilton, Canada; 6 Department of Clinical Epidemiology & Biostatistics, McMaster University, Hamilton, Canada; 7 Department of Biochemistry & Biomedical Sciences, McMaster University, Hamilton, Canada

## Abstract

Early life microbial colonization and succession is critically important to healthy development with impacts on metabolic and immunologic processes throughout life. A longitudinal prospective cohort was recruited from midwifery practices to include infants born at full term gestation to women with uncomplicated pregnancies. Here we compare bacterial community succession in infants born vaginally, with no exposure to antibiotics (n = 53), with infants who were exposed to intrapartum antibiotic prophylaxis (IAP) for Group B *Streptococcus* (GBS; n = 14), and infants born by C-section (n = 7). Molecular profiles  of the 16 S rRNA genes indicate that there is a delay in the expansion of *Bifidobacterium*, which was the dominate infant gut colonizer, over the first 12 weeks and a persistence of *Escherichia* when IAP for GBS exposure is present during vaginal labour. Longer duration of IAP exposure increased the magnitude of the effect on *Bifidobacterium* populations, suggesting a longer delay in microbial community maturation. As with prior studies, we found altered gut colonisation following C-section that included a notable lack of Bacteroidetes. This study found that exposure of infants to IAP for GBS during vaginal birth affected aspects of gut microbial ecology that, although dramatic at early time points, disappeared by 12 weeks of age in most infants.

## Introduction

The intestinal microbiota is essential for metabolic, nutritional, physiological and immunologic processes and influences a broad range of health outcomes^1,2^. In the first hours and days following birth there is a rapid evolution of microbial communities^3^ becoming adult-like before 3 years of age^4,5^. When initial exposure to the maternal microbiota is altered, for instance due to birth by Caesarean section (C-section), colonisation of the infant gut has been shown to differ in both the type and distribution of organisms^6–10^. It is possible that an atypical microbiota established early in life disrupts the initiation of metabolic and immunologic processes and contributes to lifelong changes in the host but details about microbial succession during this formative period are limited. The microbiome has been associated with chronic diseases in adulthood, such as obesity^11,12^, allergy and atopy^13^, inflammatory bowel disease^14^, and the development of colon cancer^15^, however causal effects in either direction have not been shown. It is, therefore, essential to understand infant gut microbial community succession, in the absence of medical interventions, and to study the impacts of early life exposures on the processes involved.

In North America, as many as 50% of all low risk, full term infants are exposed to intrapartum antibiotic prophylaxis (IAP) for indications such as prevention of maternal infection associated with C-section birth^16^ and management of Group B *Streptococcus* (GBS)^17^. Although treatment with IAP is considered safe, the impact on gut microbiome development in the infant is uncertain. IAP may affect non-target bacterial populations within the mother and reduce the transmission of susceptible bacterial groups to the infant during delivery. In addition, establishment of the gut microbiome may be altered as a result of IAP passed into the fetal/neonatal bloodstream through the placenta.

While many studies have addressed the gut microbiome in adults, research on the developing infant microbiome has focused mainly on the impact of C-section birth^7,9,10,18^, infant feeding practices^10,18^ and administration of oral antibiotics to infants^18,19^. Here we describe early findings of the Baby & Mi pilot study^20^, a prospective birth cohort designed to specifically determine the effects of intrapartum antibiotic administration on the development of infant gut microbiome among a low-risk population in Ontario, Canada. This longitudinal study describes the very early gut microbiome development at four time points over the first 12 weeks of life.

## Results

### Description of cohort

The Baby & Mi pilot cohort^20^ enrolled 83 mother-infant pairs. Five participants were excluded as follows: ineligibility for continued follow up due to a high risk complication prior to birth (n = 1), withdrawal prior to birth (n = 1), lost to follow up (n = 2) and not providing any samples up to the 12 week time point (n = 1). As our intent was to focus on the influence of IAP for GBS on the developing gut microbiome, we also excluded 4 participants from this analysis due to exposure to IAP for GBS and C-section (n = 3) or exposure to antibiotics for treatment of a suspected infection (n = 1). An analysis of the effect of IAP for GBS on the infant gut microbiome that also accounts for delivery mode will be undertaken in the full study, should the number of participants be sufficient to overcome the inter-individual variability in the type and abundance of shared microbial signatures. Thus, the analysed cohort was comprised of 74 mother-infant pairs. Table 1 presents the characteristics of the analysed cohort and the antibiotic exposures, stratified by mode of birth and IAP exposure. Overall, 21 women received IAP of any kind during the birth (28%): 14 (19%) received penicillin for prevention of vertical transmission of GBS during the vaginal birth; and the 7 (10%) women undergoing a C-section received IAP for prevention of infection (cefazolin (n = 5), ampicillin (n = 1) or cephalexin (n = 1)). Among the 21 women exposed to IAP the median duration of exposure was 225 minutes (95^th^ CI: 11, 831 minutes), with longer exposures in the vaginal IAP for GBS group than in those receiving IAP for a C-section. Furthermore, 63% of the vaginal IAP group were exposed to antibiotics for >4 hours prior to delivery compare to none in the C-section group. All of the C-section deliveries occurred after the onset of active labour; none were electively scheduled. All babies were breastfed at least partially. Infant exposure to antibiotics was low in the first 12 weeks of life with only 2 (3%) receiving antibiotics.

**Table 1:** Infant and maternal characteristics of the 74 mother-infant pairs.

	Vaginal birth without IAP (n = 53)	Vaginal birth with IAP (n = 14)	C-section (n = 7)
Maternal age at birth (years)	32.6 (25.5, 38.1)	32.2 (26.1, 38.2)	31.6 (27.4, 36.9)
Positive GBS status*	3 (5.66)	13 (92.9)	0 (0)
Males n(%)	27 (50.9)	8 (57.1)	3 (42.9)
Gestational age at birth (days)	282 (268, 291)	278 (270, 284)	277 (274, 290)
Birth weight (g)	3636 (3005, 4196)	3737 (3061, 4300)	3056 (2660, 5091)
**Type of intrapartum (IP) antibiotic**			
Penicillin G	—	14 (100)	0 (0)
Cefazolin	—	0 (0)	5 (71.4)
Ampicillin	—	0 (0)	1 (14.3)
Cephalexin	—	0 (0)	1 (14.3)
Duration of IP antibiotics (minutes)	—	330 (44, 849)	42 (10, 85)
IP antibiotics for ≥4 hours	—	9 (64.3)	0 (0)
Infant antibiotic use in the first 12 weeks	1 (1.89)	1 (7.14)	0 (0)

Data is presented as count (%) for categorical variables and median (5th, 95th percentile) for continuous variables. *Positive GBS status included those that had a positive screening result or documented bacteriuria.

### Effects of IAP and delivery mode on microbial communities

**Figure 1:**
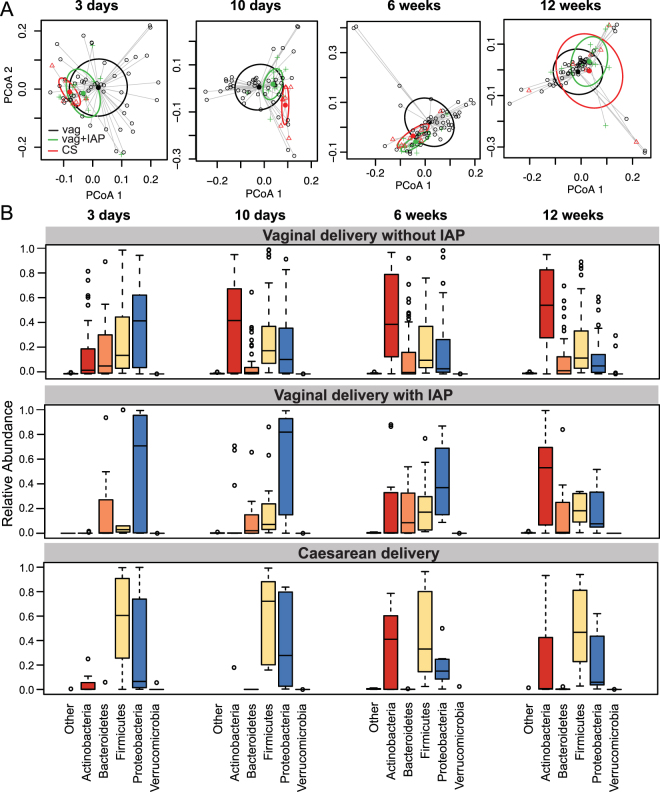
The effect of IAP exposure on microbial communities. (**A**) Beta diversity of bacterial profiles illustrated with principal coordinate analysis of Bray-Curtis dissimilarity. Samples were subset by age and IAP exposure is indicated. (**B**) Boxplots of phylum level abundance showing lower and upper quartiles along with the median value for relative abundance.

Overall, the gut microbiome of infants born vaginally without exposure to IAP differed significantly from that of infants born vaginally but exposed to IAP for GBS or born by C-section (also exposed to IAP). The bacterial community in fecal microbiota from infants exposed to IAP for GBS prior to vaginal birth, differed from that of unexposed infants at 10 days and 6 weeks of age (p < 0.05) but no differences were seen by 12 weeks (Table 2; Fig. 1A). The relative contribution of each genera to these differences is presented in Table 3. Interestingly, when assessing the impact on microbial communities of the duration of IAP exposure within vaginal births with IAP for GBS, there was a significant effect on bacterial communities at 12 weeks only (Table 2; p < 0.01), with the contribution of each genera to these differences shown in Table 4. This suggests that for most infants born vaginally, the effects of IAP on the microbiome disappear by 12 weeks of age but lasting effects remain for infants exposed to the longest course of antibiotics.

**Table 2:** Evaluating the influence of IAP, IAP duration and mode of delivery on Bray-Curtis dissimilarity matrices with Permutational multivariate analysis of variance.

Subgroup	Exposures of interest	R2	Pr(>F)
All vaginal births.			
3 days	IAP	0.032	0.07
10 days	IAP	0.053	0.011*
6 weeks	IAP	0.047	0.007*
12 weeks	IAP	0.012	0.53
**Within vaginal births with IAP for GBS**			
3 days	IAP duration	0.026	0.83
10 days	IAP duration	0.043	0.80
6 weeks	IAP duration	0.067	0.58
12 weeks	IAP duration	0.328	0.001*
**C-section vs vaginal births (without IAP)**			
3 days	Mode of delivery	0.068	0.002*
10 days	Mode of delivery	0.082	0.002*
6 weeks	Mode of delivery	0.024	0.16
12 weeks	Mode of delivery	0.045	0.024*

*p < 0.05.

**Table 3:** Difference in relative abundance of bacterial genera between IAP exposed and unexposed, vaginal births only.

△ with IAP for GBS^†^	Day 3	Day 10 (*)	Week 6 (*)	Week 12
*Bifidobacterium*	−0.063	−0.130	−0.111	−0.038
*Escherichia*	0.070	0.099	0.074	0.010
Enterobacteriaceae other	−0.008	0.059	0.030	
*Streptococcus*	0.027	−0.024	−0.012	−0.012
*Clostridium*	0.026	−0.013	0.009	0.026
Clostridiaceae other		0.020	0.017	
Lachnospiraceae other		−0.008	−0.008	−0.007
*Staphylococcus*	−0.013	−0.010		
*Bacteroides*	−0.040	0.019		0.036
*Parabacteroides*	0.041	0.005	0.006	

*IAP exposure had a significant effect on microbial profiles (p < 0.05; Table 2). ^†^Changes less than <0.005 are shown as blank.

**Table 4:** Difference in relative abundance of bacterial genera associated with each hour of IAP exposure, IAP exposed vaginal births only.

△ per hr IAP for GBS^†^	Day 3	Day 10	Week 6	Week 12 (*)
*Bifidobacterium*		−0.012	−0.032	−0.072
*Bacteroides*	0.008	0.020	0.028	0.034
*Clostridium*	−0.010			0.025
*Escherichia*	0.025	0.017		0.024
*Streptococcus*	−0.009		0.006	−0.008
*Parabacteroides*	0.010			
Enterobacteriaceae other		−0.028	−0.009	
Clostridiaceae other			0.013	

*IAP exposure had a significant effect on microbial profiles (p < 0.05; Table 2). ^†^Changes less than < 0.005 are shown as blank.

The gut microbiome of infants born by C-section was different from the microbiome in vaginally born infants, without IAP exposure, at each time point except 6 weeks (p < 0.05; Table 2; Fig. 1A). The contributions of each genera to this is presented in Table S1. No differences in the fecal microbiota of vaginally born infants exposed to IAP for GBS compared to C-section were seen in spite of the fact that these 2 groups were exposed to different intrapartum antibiotics. This last analysis, however, was likely underpowered due to the small sample size and high variability in the composition of the microbiome in infants born by C-section.

Phylum level summaries (Fig. 1B) indicate a delay in colonization with Actinobacteria in both vaginally-born infants exposed to IAP for GBS and those delivered by C-section. Further, in vaginally-born IAP for GBS exposed infants, there was a delay in colonization by Firmicutes and prolonged persistence in the abundance of Proteobacteria. In contrast, the gut microbiome in infants born by C-section lacked Bacteroidetes up to 12 weeks of age and contained a greater abundance of Firmicutes compared to infants born vaginally without exposure to IAP. These findings highlight that the pattern of the development of the gut microbiota differed between groups of infants born vaginally and those born by C-section and exposed to intrapartum antibiotics.

**Figure 2:**
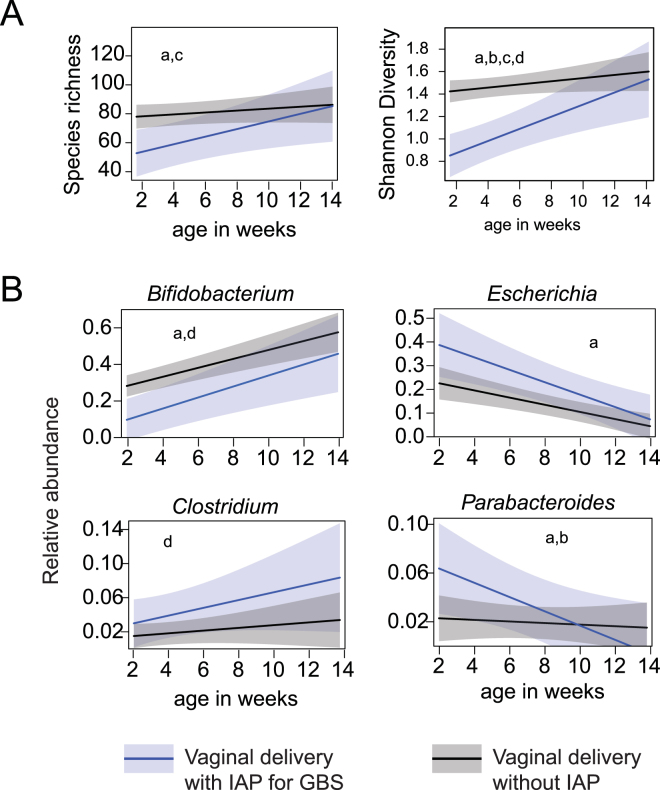
Longitudinal analysis of the effects of IAP during vaginal birth, using linear mixed models. (**A**) Alpha diversity over time of the gut microbiome in IAP unexposed and IAP exposed infants. (**B**) Abundance over time of bacterial genera significantly affected by IAP. Effects calculated with a linear mixed model that accounted for individuals over time; significance (p < 0.05) indicated as follows: (a) effect of IAP, (b) interaction of IAP and age, (c) effect of IAP duration, and d) interaction of IAP duration and age.

Bacterial species richness and Shannon diversity in the gut microbiome of infants born vaginally and exposed to IAP for GBS was significantly lower (p < 0.01) at early time points but, reached levels similar to communities in unexposed infants by 12 weeks of age (Fig. 2A). Trends could not be calculated for infants born by C-section due to the large amount of variability and the small sample size (n = 7).

### Effects of IAP and delivery mode on individual bacterial taxa

The effect of IAP exposure on the abundance of individual bacterial genera over time was assessed with a linear mixed model in order to account for individual variability. Within vaginal births, exposure to IAP for GBS had a significant negative effect on the abundance of *Bifidobacterium* (p < 0.01), a positive effect on the abundance of *Escherichia* (p < 0.05), at 12 weeks of age (Fig. 2B).

The duration of IAP for GBS exposure before birth on vaginally born infants had an effect on the overall gut microbiome of infants at 12 weeks of age. In order to speculate about which bacterial taxa might be contributing to this difference we have reported the contribution of each genus to the variability in the model (Table 4). Notably, for each hour of IAP for GBS administration during vaginal birth there was a decrease of 7.2% in the abundance of *Bifidobacterium*. This was also illustrated in Fig. 2B where the duration of IAP for GBS exposure had a negative effect on *Bifidobacterium* over time (p < 0.01) and positive effect on the abundance of *Clostridium* (p < 0.05) over time (Fig. 2B).

The abundance of several bacterial genera was associated with C-section delivery when compared with infants born vaginally and not exposed to IAP (Table S2). Notably there was a significant decrease in *Bifidobacteria, Bacteroides* and *Escherichia* in C-section born infants and an increase in unclassified genera of Enterobacteriaceae and *Clostridium* over the first 12 weeks of life.

## Discussion

Succession of bacterial populations during early infancy has an important influence on early immunological and metabolic programming that can affect long-term health^21,22^. The current study examines the succession of bacterial populations during early infancy in a healthy, full term, breastfed population while taking exposure to intrapartum antibiotics into account. We present here differences based on v3 sequencing of 16 S rRNA gene, which, although not accounting for complete species or strain level difference, illustrate the structure of the gut microbiome at these early time points. In addition to our findings supporting recent literature on the composition of the early microbiome and the influence of C-section birth (including antibiotic exposure) on the pattern of microbiome development, we made several novel observations. We identified a distinct influence of IAP for GBS on the fecal microbiota of infants born vaginally and noted that the length of intrapartum exposure to the antibiotics influenced this impact.

Similar to previous studies that found the infant gut microbiome of newborns were colonized mainly by *Bifidobacterium*, *Escherichia* and *Bacteroides* ^10,23^, we found that amongst healthy, full-term, vaginally born infants not exposed to IAP, an abundance of *Bifidobacterium* came to dominate the bacterial communities in many infants by 12 weeks, with a corresponding decrease of other members of the communities that were most dominant at 3 days and 10 days, notably *Escherichia* and *Streptococcus*.

Early life events such as preterm birth^24^, C- section^8–10,18,19^ and formula feeding^7,10,25^ have been shown to alter patterns of succession. However, prior studies have not accounted for IAP for GBS during vaginal delivery, which may have influenced these results. We have demonstrated a clear effect of IAP for GBS on the infant gut microbiome amongst vaginally born infants. The fecal microbiota of IAP exposed infants had significantly lower alpha diversity and there was a delay in the colonization patterns in these infants compared with unexposed infants. Bacterial genera most impacted by IAP for GBS during vaginal birth included *Bifidobacterium, Escherichia* and *Parabacteroides*. By 12 weeks of age, community diversity and structure of the bacterial communities of vaginally born infants exposed to IAP for GBS was similar to that of unexposed infants. However, for every hour of IAP administration, there was a decrease of 7% in the abundance of *Bifidobacterium* at 12 weeks suggesting that infants with longer IAP exposures experienced a more persistent impact. Previous studies on the effect of IAP^6^ (or GBS^26^) on the early infant gut microbiome have overlooked its impact on populations of *Bifidobacterium*. Whether this is due to underestimation of Bifidobacteria in these previous studies or a genuine difference in bacterial profiles within participants is unknown.

Birth by C-section has been associated with negative health outcomes in childhood and later life such as allergy, asthma and obesity^27,28^. Consistent with previous studies, we observed an effect of C-section delivery on the infant microbiome. Though the number of infants born by C-section was low, it is important to note that none of these babies was a planned C-section, meaning that all of the mothers had entered labour. Results from previous studies on the effect of C-section^8–10^ matched what we observed, namely an absence of Bacteroidetes for up to 12 weeks and a reduction in Actinobacteria. The reduction in the abundance of Bacteroidetes was more pronounced after C-section delivery (for which IAP is also administered) than it was after vaginal birth with IAP for GBS when compared with infants born vaginally and not exposed to IAP. This suggests that IAP exposure acts independently of mode of delivery in influencing the gut microbiota over the first 12 weeks of life. It is important to consider that the antibiotics utilized for GBS are different from those used to prevent maternal infection associated with C-section.

The first 12 weeks of life is a period marked by few outside exposures, compared to later in infancy, and is a critical period for development of the infant gastrointestinal microbiome. This study found that IAP for GBS affected all aspects of gut microbial ecology including species richness, diversity, community structure, and the abundance of colonizing bacterial genera. We found that although dramatic at early time points, effects of IAP for GBS on the microbiome during vaginal birth largely disappeared by 12 weeks of age in most infants. The impact of IAP for GBS on the gut community in infants was also impacted by the duration of IAP administration. This study recapitulated previously published observations examining the influence of C-section delivery, and suggests that IAP may act independently of mode of delivery to impact the bacterial populations that colonize the infant gut. A larger study is underway^20^ that will determine the long-term consequences of IAP for GBS on both microbial community succession and on host health and disease risk and provide the power necessary to explore in more depth the influence of maternal and infant covariates on the infant gut microbiome.

## Methods

### Study design, setting and participants

A cohort of mother-infant pairs was formed by enrolling eligible women from midwifery practices in Hamilton and Burlington, Ontario, Canada between July 1st 2012 and December 31st 2013. Women were eligible to participate in the study if they were under the care of a midwife which necessitates that they have a healthy pregnancy with low predictable risk (i.e. low risk) defined according to the Ontario Antenatal record^29^ with no pregnancy complications now or in the past, no significant maternal medical disease, no prior perinatal morbidity or mortality, with adequate fetal growth and they needed to be planning a vaginal birth. Further, they needed to be able to communicate in English. Women were excluded if they had a multiple pregnancy or a preterm birth (before 37 completed weeks gestation).

Birth data and obstetrical history, including maternal age, place of birth, mode of birth, IAP exposure (duration, dosage, type of antibiotic) and gender were collected from antenatal and birth records and mother and infant charts by the midwives. Infant stool samples and data about infant diet and infant antibiotic use were collected from the mothers as closely as possible to 3 days, 10 days, 6 weeks and 12 weeks postpartum. Diapers containing stool were transferred to a plastic storage bag, stored frozen in a household freezer and delivered to the laboratory by the women when they attended study visits with their infants at weeks 6 and 12 at McMaster University Medical Centre. Methods were carried out in accordance with the Research Ethics Boards at all participating healthcare organisations (Hamilton Health Sciences Faculty of Health Sciences (Project number: 12-201), St. Joseph’s Healthcare Hamilton (Project number: 12-3721) and Joseph Brant Hospital (Study number: 000-022-14)) and all participants, or their legal guardians, provided written informed consent. All experiment protocols were approved by McMaster University Biosafety Office (BUP-128).

### Bacterial DNA isolation and Illumina sequencing of bacterial tags

DNA was extracted from 100–200 mg of stool with a custom DNA extraction protocol involving mechanical and enzymatic lysis followed by a phenol:chloroform extraction and purification, as previously described^30^. The bacterial 16 S ribosomal ribonucleic acid (rRNA) gene v3 region (150 bp) was amplified as in^31^ and libraries were sequenced in the McMaster Genomics Facility in the forward and reverse direction on the Illumina MiSeq instrument as described previously^30^. The completed run was de-multiplexed with Illumina’s Casava software.

### Sequence processing and analysis

Illumina sequences were processed as described^30,32,39^. Briefly, after sequence trimming, alignment and filtering to remove sequences with length <100 bp, operational taxonomic units (OTU) were picked using AbundantOTU+ ^33^ with a clustering threshold of 97%. Taxonomy for each OTU’s representative sequence was assigned using the Ribosomal Database Project classifier^34^ with a minimum confidence cut-off of 0.8 against the Greengenes (2013 release) reference database. All OTUs classified as “Root:Other”, indicating that they were not of bacterial origin, were excluded.

Species number was estimated from OTU abundances with use of the rarefy function within the vegan package in R, which implements Hurlbert’s^35^ formulation for calculating the expected species richness in random subsamples from the community, calculated with a depth of 11,231 sequences. Shannon diversity was calculated with the diversity function, within the vegan package in R^36^. For principal coordinate analysis (PCoA) beta diversity was calculated with the Bray-Curtis dissimilarity measure^36^ on relative OTU abundances. For comparative analyses (permutational multivariate analysis of variance, described below) beta diversity was also calculated with the Bray-Curtis dissimilarity measure but on genus level abundances, since OTU level analysis (excluding singletons) did not indicate an effect of IAP for GBS (10 days p = 0.30, 6 weeks p = 0.08). This was likely due to inter-individual variability in OTU carriage since many OTUs provided only a minor contribution to the variability in the microbiome due to IAP for GBS exposure, whereas only a small number of genera contributed greatly to the IAP for GBS effect (Figure S1).

### Statistical analyses

Beta diversity was explored with PCoA ordination of Bray-Curtis dissimilarities calculated from relative OTU abundances. Next, a comparative analysis was done with permutational multivariate analysis of variance using Bray-Curtis dissimilarity matrices calculated from genus level taxonomic assignments with the adonis function within the vegan package in R^36^ and coefficients above 0.005 (i.e., a >0.5% change in bacterial genus level abundance between treatments) were reported in a separate table. For the comparative analysis, samples were first stratified by collection time point, then by mode of birth. Amongst vaginally born infants with IAP for GBS exposure, the effect of IAP (binary variable) and duration of IAP administration (continuous variable) were analysed. Finally, the effect of mode of birth on the microbiome was compared among Caesarean born and vaginally born infants who were not exposed to IAP for GBS.

There was a distribution of ages at each collection point (Figure S2), therefore analysis of univariate outcomes was done over time (as a continuous variable) using a generalized linear mixed effects model with infant age (in weeks) as a fixed effect and individual infant as a random effect, to account for individual variation. This was done using the lme4 package in R^37^ significance was determined with the lmerTest package in R^38^. The effect of IAP for GBS on alpha diversity estimates was modeled within vaginal births only by adding IAP to the model as a fixed effect. Abundance of each of the top 10 bacterial genera was modeled over time within infants born vaginally and either exposure to IAP for GBS or duration of IAP exposure for GBS included as a fixed effect. The effect of delivery method was similarly modeled, however, first excluding all vaginal births with IAP for GBS then including Caesarean delivery as a fixed effect. Multiple testing correction was not applied, due to the exploratory nature of this study, whose purpose was for the generation of hypotheses and not for definitive inference.

### Data availability

The datasets generated and analysed during the current study are available through NCBI SRA under accession  PRJNA403824.

### Ethics, consent and permissions

Ethics approval was obtained from the Research Ethics Boards at all participating healthcare organisations (Hamilton Health Sciences Faculty of Health Sciences (Project number: 12-201), St. Joseph’s Healthcare Hamilton (Project number: 12-3721) and Joseph Brant Hospital (Study number: 000-022-14)) and all participants provided written informed consent.

## Electronic supplementary material

Supplementary material

## References

[1] Gensollen T, Iyer SS, Kasper DL, Blumberg RS (2016). How colonization by microbiota in early life shapes the immune system. Science.

[2] Belkaid Y, Hand TW (2014). Role of the microbiota in immunity and inflammation. Cell.

[3] Palmer C, Bik EM, DiGiulio DB, Relman DA, Brown PO (2007). Development of the human infant intestinal microbiota. PLoS Biol..

[4] Koenig JE (2011). Succession of microbial consortia in the developing infant gut microbiome. Proc. Natl. Acad. Sci. USA.

[5] Yatsunenko T (2012). Human gut microbiome viewed across age and geography. Nature.

[6] Azad MB (2016). Impact of maternal intrapartum antibiotics, method of birth and breastfeeding on gut microbiota during the first year of life: a prospective cohort study. BJOG.

[7] Penders J (2006). Factors influencing the composition of the intestinal microbiota in early infancy. Pediatrics.

[8] Madan, J. C. *et al*. Association of Cesarean Delivery and Formula Supplementation With the Intestinal Microbiome of 6-Week-Old Infants. *JAMA Pediatr*. 1–8 (2016). 10.1001/jamapediatrics.2015.3732 PMC4783194 26752321

[9] Dominguez-Bello MG (2010). Delivery mode shapes the acquisition and structure of the initial microbiota across multiple body habitats in newborns. Proc. Natl. Acad. Sci. USA.

[10] Bäckhed F (2015). Dynamics and Stabilization of the Human Gut Microbiome during the First Year of Life. Cell Host Microbe.

[11] Ley RE (2005). Obesity alters gut microbial ecology. Proc. Natl. Acad. Sci. USA.

[12] Finucane MM, Sharpton TJ, Laurent TJ, Pollard KS (2014). A taxonomic signature of obesity in the microbiome? Getting to the guts of the matter. PLoS One.

[13] Penders J (2013). Establishment of the intestinal microbiota and its role for atopic dermatitis in early childhood. J. Allergy Clin. Immunol..

[14] Knights D, Lassen KG, Xavier RJ (2013). Advances in inflammatory bowel disease pathogenesis: linking host genetics and the microbiome. Gut.

[15] Irrazábal T, Belcheva A, Girardin SE, Martin A, Philpott DJ (2014). The multifaceted role of the intestinal microbiota in colon cancer. Mol. Cell.

[16] van Schalkwyk J, Van Eyk N (2010). Society of Obstetricians and Gynaecologists of Canada Infectious Diseases Committee. Antibiotic prophylaxis in obstetric procedures. J. Obstet. Gynaecol. Can..

[17] Spaetgens R (2002). Perinatal antibiotic usage and changes in colonization and resistance rates of group B streptococcus and other pathogens. Obstetrics & Gynecology.

[18] Bokulich NA (2016). Antibiotics, birth mode, and diet shape microbiome maturation during early life. Sci. Transl. Med..

[19] Yassour M (2016). Natural history of the infant gut microbiome and impact of antibiotic treatment on bacterial strain diversity and stability. Sci. Transl. Med..

[20] Julia Simioni, Eileen K Hutton, Elizabeth Gunn, Alison Holloway, Jennifer Stearns, Helen McDonald, Andrea Mousseau, Jonathan Schertzer, Elyanne Ratcliffe, Lehana Thabane, Michael Surette, Katherine M Morrison. A Comparison Of Intestinal Microbiota In A Population Of Low Risk Infants Exposed And Not Exposed To Intrapartum Antibiotics: The Baby & Microbiota Of The Intestine Cohort Study Protocol (2016). 10.1186/s12887-016-0724-5 PMC5103394 27832763

[21] Dietert, R. R. The microbiome-immune-host defense barrier complex (microimmunosome) and developmental programming of noncommunicable diseases. *Reprod. Toxicol*. 10.1016/j.reprotox.2016.04.026 (2016). 10.1016/j.reprotox.2016.04.026 27167696

[22] Wallace JG, Gohir W, Sloboda DM (2016). The impact of early life gut colonization on metabolic and obesogenic outcomes: what have animal models shown us?. J. Dev. Orig. Health Dis..

[23] Vatanen T (2016). Variation in Microbiome LPS Immunogenicity Contributes to Autoimmunity in Humans. Cell.

[24] DiBartolomeo, M. E. & Claud, E. C. The Developing Microbiome of the Preterm Infant. *Clin. Ther*., 10.1016/j.clinthera.2016.02.003 (2016). 10.1016/j.clinthera.2016.02.003 PMC4851874 26947798

[25] Victora CG (2016). Breastfeeding in the 21st century: epidemiology, mechanisms, and lifelong effect. Lancet.

[26] Cassidy-Bushrow AE (2016). Maternal group B Streptococcus and the infant gut microbiota. J. Dev. Orig. Health Dis..

[27] Thavagnanam S, Fleming J, Bromley A, Shields MD, Cardwell CR (2008). A meta-analysis of the association between Caesarean section and childhood asthma. Clinical & Experimental Allergy.

[28] Blustein J (2013). Association of caesarean delivery with child adiposity from age 6 weeks to 15 years. Int. J. Obes..

[29] Ontario Ministry of Health and Long-Term Care. OMA Antenatal Record 1 (2005).

[30] Stearns JC (2015). Culture and molecular-based profiles show shifts in bacterial communities of the upper respiratory tract that occur with age. ISMEJ.

[31] Bartram AK, Lynch MDJ, Stearns JC, Moreno-Hagelsieb G, Neufeld JD (2011). Generation of multimillion-sequence 16S rRNA gene libraries from complex microbial communities by assembling paired-end illumina reads. Appl. Environ. Microbiol..

[32] Whelan FJ (2014). The loss of topography in the microbial communities of the upper respiratory tract in the elderly. Ann. Am. Thorac. Soc..

[33] Ye, Y. Identification and quantification of abundant species from pyrosequences of 16S rRNA by consensus alignment. In *2010 IEEE International Conference on Bioinformatics and Biomedicine (BIBM)* 153–157 (2010). 10.1109/BIBM.2010.5706555 PMC3217275 22102981

[34] Wang Q, George MG, Tiedje JM, Cole JR (2007). Naive Bayesian Classifier for Rapid Assignment of rRNA Sequences into the New Bacterial Taxonomy. Appl. Environ. Microbiol..

[35] Hurlbert SH (1971). The Nonconcept of Species Diversity: A Critique and Alternative Parameters. Ecology.

[36] Oksanen, J. *et al*. *vegan: Community Ecology Package* (2015).

[37] Bates, D., Maechler, M., Bolker, B. M. & Walker, S. Fitting Linear Mixed-Effects Models using lme4 (2015).

[38] Kuznetsova, A., Bruun Brockhoff, P. Haubo Bojesen Christensen, R. *lmerTest: Tests in Linear Mixed Effects Model*s (2015).

[39] Whelan, F.J. & Surette, M.G. A comprehensive evaluation of the sl1p pipeline for 16S rRNA gene sequencing analysis. *Microbiome* 2017 Aug 14;5(1):100. 10.1186/s40168-017-0314-2 PMC5557527 28807046

