# Supplementary material for: Intrapartum antibiotics for GBS prophylaxis alter colonization patterns in the early infant gut microbiome of low risk infants
Source: Sci Rep. 2017 Nov 28;7:16527. doi: 10.1038/s41598-017-16606-9 (PMC5705725; doi:10.1038/s41598-017-16606-9)
Supplement: Supplementary file 1 — Supplementary material [file 41598_2017_16606_MOESM1_ESM.pdf]

Supplemental Figures

Intrapartum antibiotics for GBS prophylaxis alter colonization patterns in the early infant gut microbiome of low risk infants

Jennifer C. Stearns, Julia Simioni, Elizabeth Gunn, Helen McDonald, Alison C. Holloway, Lehana Thabane, Andrea Mousseau, Jonathan D. Schertzer, Elyanne M. Ratcliffe, Laura Rossi, Michael G. Surette, Katherine M. Morrison and Eileen K. Hutton

Supplemental Figure 1: Histogram of the magnitude of each coefficient contributing to the effect of IAP exposure on the infant gut microbiome at 6 weeks, when permutational multivariate analysis of variation was done at A) the genus level and B) the OTU level.

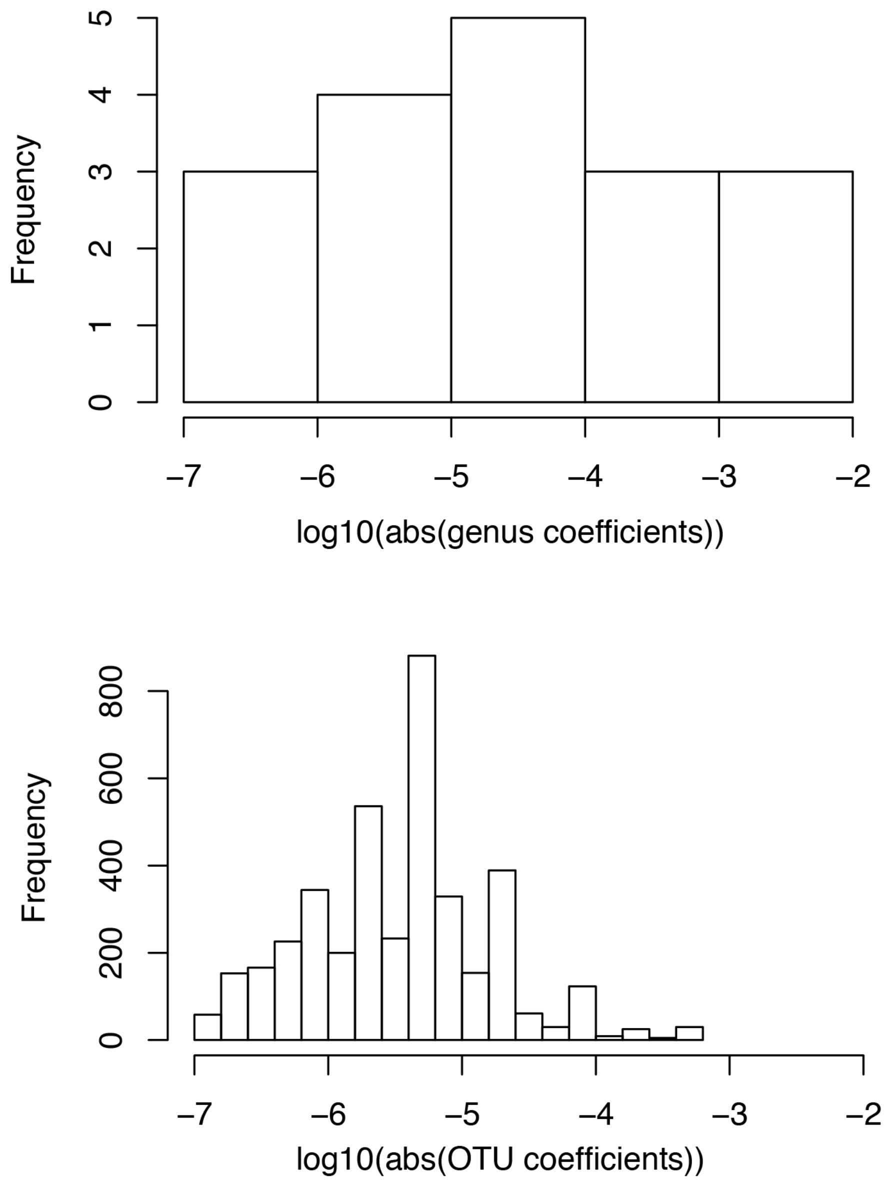

Supplemental Figure 2: Distribution of infant ages in weeks represented within this study.

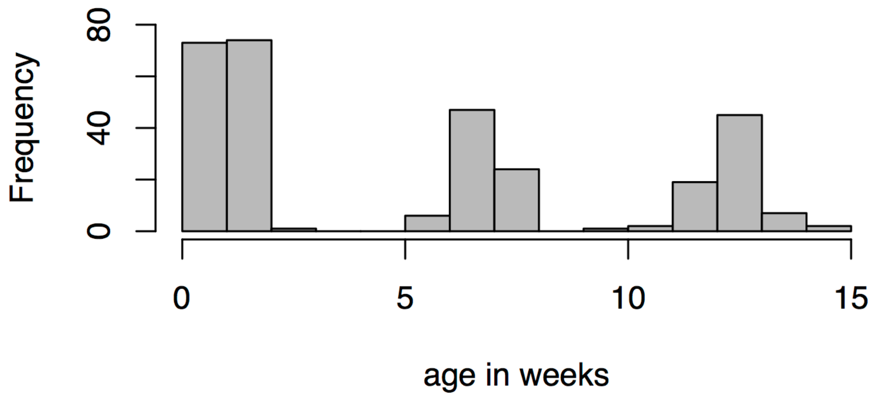

Table S1: Change in relative abundance of bacterial genera associated with Caesarean delivery compared with vaginal births unexposed to IAP.

**△ with C-section birth compared to vaginal birth without IPA †**	**day 3 (*)**	**day 10 (*)**	**week 6**	**week 12 (*)**
Bifidobacterium [a]	-0.047	-0.197	-0.042	-0.143
(Lachnospiraceae) *Ruminococcus* [b]	-0.009	0.011	-0.011	0.046
Bacteroides [a]	-0.067	-0.027	-0.054	-0.045
Lachnospiraceae other	0.021	0.009	0.040	0.045
Enterobacteriaceae other [a]	0.101	0.066	0.037	0.043
Clostridium [a]		0.042	0.051	0.038
Streptococcus	0.019	-0.015		-0.017
(Lachnospiraceae) *Epulopiscium* [a,b]				0.015
Escherichia [a,b]	-0.160		-0.031	0.014
Clostridiaceae other [a]		0.064	0.020	0.012

* Delivery by Caesarean had a significant effect on microbial profiles ($p < 0.05$)

† Changes less than < 0.005 are shown as blank.

[a] Linear mixed model accounting for individual infant over time indicated a significant effect ($p < 0.05$) of Caesarean delivery on the abundance of these genera,

[b] Linear mixed model accounting for individual infant over time indicated a significant ($p < 0.05$) interaction effect between Caesarean delivery and infant age on the abundance of these genera.
